# Women’s sexual and reproductive health in war and conflict: are we seeing the full picture?

**DOI:** 10.1080/16549716.2023.2188689

**Published:** 2023-03-17

**Authors:** Jenny Hedström, Tobias Herder

**Affiliations:** a Department of War Studies and Military History, Swedish Defence University, Stockholm, Sweden; b Division of Social Medicine and Global Health, Department of Clinical Sciences, Lund University, Malmö, Sweden

**Keywords:** SRHR, war, gender, Myanmar, reproductive health

## Abstract

It is well established that women’s sexual and reproductive health (SRHR) is negatively affected by war. While global health research often emphasises infrastructure and systematic factors as key impediments to women’s SRHR in war and postwar contexts, reports from different armed conflicts indicate that women’s reproduction may be controlled both by state and other armed actors, limiting women’s choices and access to maternal and reproductive health care even when these are available. In addition, it is important to examine and trace disparities in sexual reproductive health access and uptake within different types of wars, recognising gendered differences in war and postwar contexts. Adding feminist perspectives on war to global health research explanations of how war affects women's sexual and reproductive health might then contribute to further understanding the complexity of the different gendered effects war and armed conflicts have on women’s sexual and reproductive health.

## Women’s reproductive health in war and conflict

It is well established that reproductive insecurities contribute to higher mortality for women during both war and postwar periods [1–4], and add to women’s exclusion from decision-making on issues concerning peace and conflict [5]. Reproductive insecurities also contribute to women’s poverty [6], with potential harmful effects for sustainable development in postwar and conflict-affected contexts. This suggests that improving reproductive health has possible long-term societal benefits, by both improving women’s position and health, and by reducing poverty and inequality in decision-making [7]. Recognising this, the UN Sustainable Development Goals situate maternal health as a global priority, and recent resolutions in the Women, Peace and Security agenda stresses sexual and reproductive health assistance for survivors of sexual violence.

However, we still do not know enough about how and why women’s sexual and reproductive health and rights (SRHR) are affected by wars and armed conflicts.

Existing research in global health often emphasise health infrastructure as the key factor affecting women’s and girls’ health in war and postwar settings. Studies show that organised violence increases the risk of maternal deaths because of the breakdown or reduction of health infrastructure, constraining women’s and girls’ access to reproductive and maternal health services [8]; because of structural and secondary factors such as malnutrition, poverty and a lack of clean water [9]; and sexual and gender-based violence, and other human rights violations [10,11]. We know now that restricted access to family planning and health care initiatives lead to an increase in unwanted pregnancies and unsafe abortions [8,12], with research suggesting that interventions focused on improving access to maternal health can offset the negative effects of war on women’s and girl’s wellbeing [13] through, for example, increasing the availability of maternal and reproductive health services [14], clean water and nutrition [11] and/or peacekeeping operations providing security [13]. In other words, this body of research has in important ways helped increase recognition of the impact of war on women’s reproductive health and rights by demonstrating 1) that wars impede access to maternal health care; and 2) that this can be offset by targeted interventions focused on increasing access to and availability of health care; contraceptives; and essential nutrients. While these data have extended our understanding of women’s reproductive health in war in critical ways, they remain yoked to explanations privileging infrastructure and systematic factors impacting SRHR in war and postwar situations. As such, they do not provide a complete picture of how and why SRHR are impacted by war.

This is because women and girl’s bodily integrity and reproductive security is a phenomenon both deeply politicised as well as securitised. We suggest that applying feminist perspectives on war to global health research concerning SRHR in wars can help draw our attention to how women’s bodies are at the centre of policies and violent struggles that have variously been described as a form population control [15]; genocide [16]; ethnic cleansing [17]; and as a weapon of war [18]. Furthermore, studies show that rebel groups monitor reproductive policies and relations between recruits, expecting recruits to retire upon pregnancy or marriage [19,20], impose abortions or contraceptive use on women [21] and enforce marriages between soldiers or between soldiers and local women [22]. Recent reports from Nigeria suggest that Boko Haram forcibly married and raped women and girls; in turn, the state military enforced abortions on women released from the non-state armed group [23]. Controlling and adapting practices and policies around conjugal relations and reproduction to fit military objectives appears to be a crucial aspect of war, suggesting that SRHR is not incidental to war but integral to military tactics and strategies, shaping women’s experiences of both war and postwar periods.

## The example of Myanmar

To illustrate the complex relationship between war and women’s sexual and reproductive health, we can use the case of Myanmar. Myanmar has a long history of ethno-nationalistic wars, wherein women’s bodies and reproduction have been policed, monitored and utilised as a strategy of warfare and population control by both state and non-state militaries [24]. Research from Myanmar suggests that the history of ethnic armed conflict, as well as the military organisations themselves, shape reproductive policies, and in particular contraceptive uptake, in multiple ways. This research illustrates how ethnic non-state military actors interrupt family planning initiatives [25], discouraging contraceptive use [26], asking women to ‘reproduce for the revolution’ [20], in order to counter what is widely perceived as attempts by the state to eradicate and ‘dilute’ ethnic minority communities. Birth control is often understood as a form of population control aimed at ethnic minority communities, with recent legislation restricting the number of births minority women can have and who they can marry [27]. Decades of war, followed by brutal counterinsurgency campaigns aimed at ethnic minority communities and the re-routing of funding from healthcare to military needs, seems to have severely hampered health infrastructure in general and women’s access to reproductive health services in particular. Indeed, recent data show that women living in ethnic minority regions are both least likely to use modern contraceptives [28] and have higher estimates of maternal mortality as compared to those living in non-ethnic areas [29]. This is concerning, as Myanmar has exceptionally high levels of maternal mortality and morbidity overall, with 10% of maternal deaths being attributed to unsafe abortions [30]. Yet reports also show that Myanmar scores relatively high on the Sustainable Development Goals family planning indicators [31] and that awareness among women for family planning is widespread [32]. What can then explain the low uptake [28] and high maternal mortality in ethnic minority communities [29]?

## Seeing the full picture

The question of how and why women’s sexual and reproductive health matter in wars thus remain theoretically and empirically underexplored, highlighting the need to pay closer attention to this relationship. Moreover, previous studies have subsumed very different types of wars under one heading. While some exceptions to this exist [see [33]], a large proportion of research in global health approach conflict as one phenomenon rather than as a complex and varied political phenomenon with complex and varied gendered effects. For example, studies on maternal health care use in the Democratic Republic of Congo [14,34], Cameroon, Mali, and Nigeria; and Africa [35,36] as well as globally [37,38] take the primary definition of war from the UCDP dataset as given. However, different types of wars have different effects, and it is important to carefully identify and analyse these effects across and within conflicts. For instance, regular wars will use different methods, tactics and strategies as compared to irregular or hybrid wars [39]. Ethnic conflict, revolutionary uprisings, genocide, terrorist attacks and civil wars are all strategized, fought and experienced differently on the ground. Thus, rather than lumping arguably very different wars together as one, it is important to carefully unpack, examine and trace disparities in reproductive health access and uptake within different types of conflicts. Indeed, feminist research on sexual violence against women in war demonstrates how sexual and other forms of gender-based violence ‘take various forms and exhibit different patterns across contexts’ [40], showing extensive regional variation, both across and within wars [41–43]. Moreover, armed groups, even those fighting in the same region, may exhibit vastly different policies and practices regarding women’s sexual and reproductive health and rights. For example, in Myanmar, while the Restoration Council of Shan State has prevented public health workers from carrying out family planning initiatives [25], others, like the Kachin Independence Organisation have allowed for this work within their communities (see kachinwomen.com).

Existing research thus shows that first, SRHR are not incidental to but an integral part of military strategies and the conduct of wars, and second, affect women’s vulnerability and mortality in war beyond the immediacy of the battlefield. Third, analysis of SRHR in war needs to be undertaken at the micro rather than at the macro level to allow for granular analysis of why and where these rights are recognised and why and where they ignored or outright abused. While insights and methods from public health can help us to identify and examine the relationship between conflicts and their effects on women, feminist perspectives on wars could help us explore the varied ways in which these dynamics are experienced, understood and received on the ground. Moreover, these insights are essential to guide future interventions to strengthen reproductive health in conflict setting, as evidence of effective interventions today are scarce [4].

While the most common and, in the words of Jürg Utzinger and Mitchell Weiss ‘obvious way’ to consider public health implications of war is in terms of civilian and military casualties and injuries [44], a focus on direct mortality risks obfuscating the more long-term and gendered effects of armed conflicts which affects the bodies of women and girls in uneven ways. In a new global landscape with emerging polarisation, conflicts and war, the collection of further data and theorising on why this is the case appear urgent. Here, listening to and learning from women and girls living in war and postwar settings is crucial for making visible the interlocking hierarchies of gendered power producing and obscuring reproductive harms long after the official end of conflict.

We suggest that studies in global health could benefit from merging insights from public health and feminist war studies, enabling not only explanations on how war affect women and girls’ sexual and reproductive health, but also allowing us to respond to the fundamental question: why? Why are women and girl’s sexual and reproductive rights violated and manipulated in armed conflict, and how can we prevent or resolve war’s effects on the bodily integrity and wellbeing on women and girls? By acknowledging the complexity of wars and armed conflicts, and the different gendered effects it has on women’s sexual and reproductive health, we can get closer to seeing the full picture and understand the ‘why’ - which is a first and essential step for action to protect women’s SRHR.

## Paper context

Global health research on sexual and reproductive health and rights in wars and armed conflicts tend to emphasise disruptions of infrastructure with negative effects on access to services as the main challenge. Adding feminist perspectives on war to global health research explanations of how war affects women’s and girls’ sexual and reproductive health would acknowledge the complexity of wars and armed conflicts, and the different gendered effects it has on women’s sexual and reproductive health.
